# Empowering Indigenous natural hazards management in northern Australia

**DOI:** 10.1007/s13280-022-01743-x

**Published:** 2022-06-27

**Authors:** Jeremy Russell-Smith, Glenn James, Alan Maratja Dhamarrandji, Ted Gondarra, Danny Burton, Bevlyne Sithole, Otto Bulmaniya Campion, Hmalan Hunter-Xenie, Ricky Archer, Kamaljit K. Sangha, Andrew C. Edwards

**Affiliations:** 1 Darwin Centre for Bushfire Research (DCBR), Charles Darwin University, Darwin, NT 0909 Australia; 2 Bushfire & Natural Hazards Cooperative Research Centre (BNHCRC), East Melbourne, Victoria Australia; 3 North Australian Indigenous Land & Sea Management Alliance Ltd (NAILSMA), Darwin, NT Australia; 4 Dalkarra and Djirrikay Authority (DDA), Galiwin’ku, NT Australia; 5 Aboriginal Research Practitioners Network (ARPNet), Darwin, NT Australia

**Keywords:** Climate change, Indigenous people, Natural disasters, Participatory action research, Risk management, Vulnerable communities

## Abstract

Northern Australia is prone to recurring severe natural hazards, especially frequent cyclones, flooding, and extensive wildfires. The region is sparsely populated (≪ 0.5 persons km^−2^), with Indigenous (Aboriginal) residents comprising 14% of the population, and typically the majority in remote regions. Despite national policy committed to addressing emergency management (EM) in vulnerable Indigenous communities, implementation remains unfunded. We synthesise participatory intercultural research conducted over seven years exploring core challenges, opportunities and potential solutions towards developing effective EM partnerships. Similar EM engagement and empowerment issues face First Nations and local communities in many international settings. In search of solutions, we explore developing effective partnership arrangements between EM agencies and culturally diverse Indigenous communities. Observing that government already provides substantial investment in cultural and natural resource management programmes conducted by over 150 Indigenous Ranger Groups (IRGs) nationally, we demonstrate that expansion of IRG roles to incorporate EM community engagement and service delivery can provide multiple cost-effective community and business development benefits for many remote communities.

## Introduction

Australia faces recognised increasing climate change risks, impacts and costs associated with natural hazards including floods, cyclones, storms, bushfires, heatwaves, earthquakes and tsunamis (DHA 2018). At a political and demographic level, these emerging risks and costs particularly affect the relatively densely populated social, natural, built and economic environments of coastal and sub-coastal regions of south-eastern and -western Australia. For Australia’s sparsely settled monsoonal to arid rangelands, covering 81% of Australia’s 7.9 M km^2^ landmass and supporting 1.7% of the population (ABS 2016; Foran et al. 2019), projected climate change impacts over the next 50 years include significant increases in temperature and climatic variability (CSIRO 2020), with predicted critical impacts on agriculture, natural resources, regional and predominantly Indigenous remote communities and human distress (Cleugh et al. 2007; McKeon et al. 2009). By contrast with recent decline in the non-Indigenous rangelands population, the Indigenous population increased by 5.1% between 2011 and 2016 to comprise 27.8% of the total regional population (ABS 2016; Foran et al. 2019). Despite Indigenous Australians now having very substantial interests in and title to their ancestral lands in remote Australia (Archer et al. 2019; Sangha et al. 2019a), Indigenous people continue to live under conditions of severe social, cultural and economic disadvantage (CoA 2020).

In sparsely populated northern Australia, remote communities are at risk from natural hazards including frequent wet season cyclonic and flooding events, and extensive (if of relatively low intensity) annual dry season savanna wildfires and the smoke these produce. These hazards are projected to increase significantly in the decades ahead; for example, the number of very hot (> 35 °C) days per year in parts of northern Australia is projected to increase from 20 currently to approximately 250 by the end of century (Moise et al. 2015). Most regional remote communities typically possess limited, if any, emergency infrastructure resources (e.g. cyclone shelters; fire-fighting equipment) and structural capacities (e.g. all-season logistical access to communities; accessible EM plans; appropriate training; informed local governance) to mitigate against, prepare for, and respond to such events (Howitt et al. 2012; Sangha et al. 2017a, b).

Australian Government policy concerning EM arrangements in remote Indigenous communities is espoused in the *Keeping our mob safe* strategy (CoA 2007a), which, as discussed later, describes the primacy of developing effective partnerships between remote Indigenous community governance structures and EM agencies, including co-development of community EM plans. A subsequent Council of Australian Governments’ review recognised that *"improved disaster management outcomes in remote Indigenous communities will only be achieved if the associated systems and structures are informed by the cultural needs and perspectives of those communities*” (COAG 2011). The same review also identified key vulnerabilities that affect the capacity of remote communities to deal effectively both with major natural hazards and longer-term resilience: critical population mass; serviceable community infrastructure; accessible services; sustainable local economies; literacy and numeracy; community governance. The national remote Indigenous community EM strategy remains unfunded to the present day.

Conversely, little recognition typically has been given in Australia to core social capital values contributing to community resilience afforded by Indigenous kinship systems and networks, traditional knowledges and local governance institutions (Ellemor 2005; Petheram et al. 2010; Veland et al. 2010; Howitt et al. 2012; Zurba et al. 2012). Similarly, issues concerning the importance of, and lack of formal recognition given to, effective engagement with Indigenous knowledges and associated cultural institutions is widely acknowledged in international EM, disaster risk reduction, community resilience and emerging climate change contexts (e.g. Berkes 1999; Turner and Clifton 2009; Mercer et al. 2010; Kenney and Phibbs 2014; Rahman et al. 2017; Whyte 2017; Stacey et al. 2019; Yumagolova et al. 2021).

In this paper, we present a critical assessment of the status of natural disaster preparedness and emergency management arrangements, challenges and opportunities, as these relate to remote Indigenous communities with a particular focus on coastal and hinterland regions of monsoonal northern Australia. Research reported here synthesises seven years’ intercultural collaborative socio-environmental and policy research broadly addressing the theme, *Building capacity in north Australian remote communities*, funded through Australia’s national Bushfires and Natural Hazards Cooperative Research Centre. That programme has focussed on: (1) understanding the particular EM challenges faced by, and aspirations of, remote Indigenous communities as perceived by community members themselves (NAILSMA 2014; Morley et al. 2016; Lawurrpa et al. 2017; Sangha et al. 2017b; Sithole et al. 2017, 2019a, b, 2021; James et al. 2019, 2021); (2) exploring sustainable ecosystem services and economic opportunities for supporting Indigenous engagement in effective EM partnerships (Sangha et al. 2017a, 2019a, b, c, 2020, 2021; Russell-Smith and Sangha 2018, 2019; Russell-Smith et al. 2019, 2020a, b).

In sections following we consider (i) the geographical and demographic context of natural hazards and remote communities in northern Australia; (ii) the roles of EM agencies and institutions for delivery of effective EM arrangements; (iii) the expectations and aspirations of remote communities towards developing effective EM partnerships; and (iv) exploring solutions for developing effective EM arrangements in remote communities, and realising the long-term benefits, including financial, associated with empowerment of remote community resilience.

## Geographic and demographic context of natural hazards and remote communities in northern Australia

**Fig. 1:**
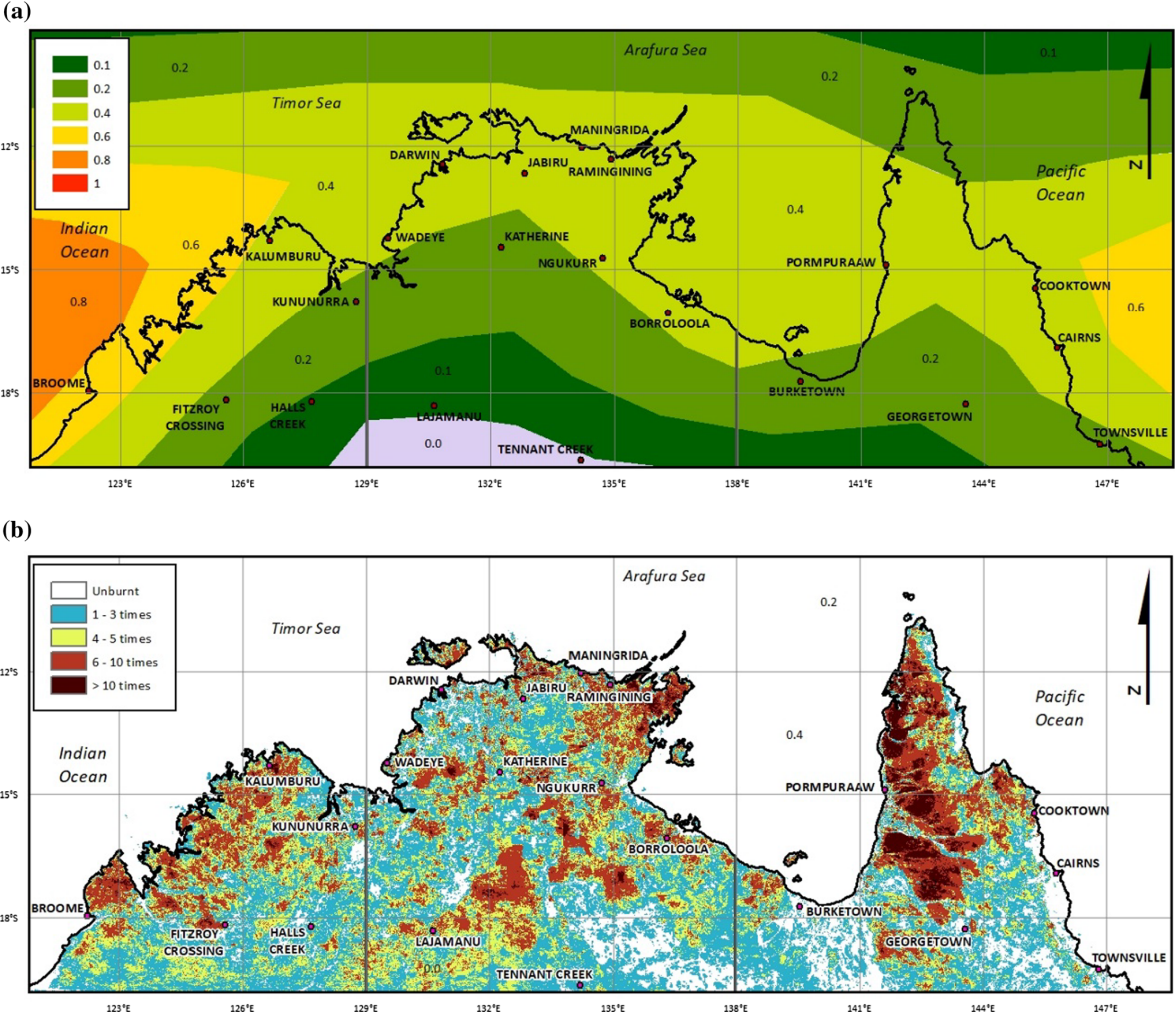
**a** Tropical Cyclone frequency, 1969–2018 ( Source: http://www.bom.gov.au/climate/maps/averages/tropical-cyclones/), **b** frequency of annual wildfires (i.e. Late Dry Season fires—1 Aug to 31 Dec), 2000 to 2020 (Source: http://www.firenorth.org.au)

Any location in north Australia, defined here as the region north of − 20° S, is prone to recurring severe cyclonic and associated flooding events on average every second year (Fig. 1a). Over the period 2010–2021, 79 cyclones have impacted the north Australia region, including 17 Severe Tropical Cyclones (Categories 3–5; sustained winds > 165 kph) with landfall (Source: Bureau of Meteorology, http://www.bom.gov.au/cyclone/tropical-cyclone-knowledge-centre/history/past-tropical-cyclones/). Under predicted climate change effects, peak winds in cyclones will increase by 5–10% and peak rainfall rates rise by 20–30% (CoA 2009). Additionally, many remote communities are cutoff to trafficable road access each wet season (Nov–Apr) given seasonal flooding for months at a time. At such times, ready access is restricted to transport in small planes and the availability of a runway.

The northern savannas region is also annually prone to late dry season wildfires (Fig. 1b). Although savanna fires are characteristically ground-borne and of relatively low severity by comparison with canopy-borne fires in southern Australian eucalypt forests and woodlands (Gill et al. 1981; Burrows and McCaw 2013), in the absence of built (e.g. roads) or natural (e.g. watercourses) barriers, late dry season (LDS) savanna fires commonly burn over very extensive (e.g. ≫ 1000 km^2^) areas consuming all available ground fuels, impacting natural and cultural values, and putting communities and infrastructure at significant risk (Yates et al. 2008; Russell-Smith et al. 2020a). Building on a long tradition of Indigenous landscape-scale fire management practice, regional contemporary practice focuses on the implementation of prescribed burning in early dry season (EDS) months (generally prior to August) to reduce ground fuels and mitigate risk (Dyer et al. 2001).

The regional population density and distribution of north Australian communities is located mostly in cyclone-vulnerable coastal and sub-coastal areas. Of 126 communities north of − 20° S with > 200 residents, 69 have populations with a majority of Indigenous residents—including 51 with < 1000 residents, and 18 with between 1000 and 10 000 residents. The North Australia population, including major towns, comprised ~ 1 million people in the 2016 census, of whom 14% were Indigenous. Excluding major town centres, the North Australia savanna population was around 170 000, at an average population density of 0.14 persons.km^−2^. In the Kimberley (Western Australia) and Top End (Northern Territory) savannas, about half of the population is Indigenous, and in very remote regions generally, more than 90% (Taylor 2006).

For Australia as a whole, the gross value of natural hazards-related, mostly tangible losses have been estimated to vary between AUD 1.36 and 19.46B per yr (Deloitte Access Economics 2016, 2017; Handmer et al. 2018; Ladds et al. 2017). In northern Australia, major cyclonic events are associated with most structural damage, especially in relatively densely populated parts of north-east Queensland where Cyclone Yasi (2011) caused structural damage worth AUD 1.41B, Cyclone Marcia (2015) AUD 544 M and Cyclone Larry (2006) AUD 540 M (Insurance Council of Australia 2021). In the Northern Territory, with a much sparser remote population density, closely occurring Cyclones Lam and Nathan in 2015 caused AUD 45 M and AUD 30 M structural losses (Insurance Council of Australia 2021; Finity Consulting 2015), albeit to just a few small remote communities (see Boxes 1, 2). Estimated total structural costs from major natural hazard events in Australia are anticipated to reach AUD 39B by 2050, even in the absence of considering climate change impacts or environmental losses (Deloitte Access Economics 2017).

Typically, associated environmental costs incurred by natural hazards are not accounted for. However, for the Northern Territory, Sangha et al. (2021) estimated natural hazard-related environmental costs where, on average, cyclone-related losses were worth AUD 17 M per event or AUD 11.5 M per yr (using a 10 year average from 2010 to 2019), and flooding losses varied from ~ AUD 1 M up to AUD 3 M per event, or up to AUD 7.3 M if a monsoon trough was involved. Loss of ecosystem services from large (> 100 km^2^) wildfires was estimated at AUD 95 M per yr and cyclones AUD 7.6 M per yr (Sangha et al. 2021). From a local Indigenous community context, on-ground tangible costs from natural hazards for a typical remote Northern Territory community were calculated at AUD 3.8 M per yr (Sangha et al. 2021). Across northern Australia, there are more than 200 remote Indigenous communities facing multiple natural hazards annually. Losses to cultural assets and impacts on social/spiritual well-being, though not ascribed monetary value, are considered critical by customary land owners.

## Formal Emergency Management arrangements

### The roles of EM agencies and institutional barriers to delivery of effective EM arrangements

Emergency Management Australia (EMA) is the peak body guiding State and Territory governments, non-government organisations (NGOs), EM organisations and agencies and communities, for addressing emergency management arrangements in Australia (AIDR 2019). EMA articulates the principles, structures and procedures that support national coordination of EM under the EMA arrangements, mainly through the *National Strategy for Disaster Resilience* (COAG 2011). However, each State and Territory also has its own legislation for dealing with emergencies and disasters.

The *National Strategy* provides a rationale for governments to focus on prevention, mitigation, preparedness and building capability, to enhance resilience to emergencies across Australia. In April 2018, a collaborative approach was adopted by the Australian Government to apply the international *Sendai Framework for Disaster Risk Reduction 2015–2030*, which recognises not only the importance of managing disasters but also risks (AGDHA 2018), with the launching of a comprehensive ‘*National Disaster Risk Reduction Framework*’ setting out domestic policies for reducing natural disaster-related risks through to 2030 (AGDHA 2018; AIDR 2019).

At the State/Territory level, each jurisdiction has their own fire and EM agencies responsible to mitigate, manage and respond to emergencies including storms and floods, cyclones and bushfires (Table 1). In addition, key Non-Government organisations such as Red Cross play a vital role in managing and supporting emergency response situations. Current EM arrangements across all northern jurisdictions support major regional towns and cities but tend to focus only on immediate response and recovery to major natural disasters in larger remote communities (Sangha et al. 2019b). EM delivery by agencies is heavily reliant on volunteer units (Table 1) based in and around major urbanised centres—few operational volunteer units are available to service more remote Indigenous communities, either for responding to critical if periodic flooding and cyclonic events or to assist with annual fire mitigation activities (Sangha et al. 2019b; Russell-Smith et al. 2020a).

**Table 1:** Key organisations and their responsibilities to manage bushfires and emergencies across northern Australia

State/territory Key organisations	Responsibilities	Relevant legislation
Northern Territory^a^		
*Bushfires NT* (No. of employees: ~ 30; active volunteers 500)	To implement measures for bushfire mitigation, management and suppression in non-urban areas, mainly to help protect life, property and the environment from bushfires	Bushfires Management Act 2016 (Act no. 14 of 2016)
NT Fire and Rescue Services (NT FRS), and NT Emergency Services (NTES): (No. of employees ~ 200 in NTFRS, and 10 in NTES; overall > 1500 including police; and > 500 volunteers)	To manage fire in mainly in urban areas (NTFRS), and to undertake risk reduction, prevention, preparation for, respond to and recover from emergencies – NTES/NTFRS NTES is mainly responsible to manage emergencies such as cyclones, storms and floods across the territory, whilst NTFRS for fire mainly in urban jurisdictions	Fire and Emergency Act 1996 (NT) (current from 1 November 2016)
Queensland^b^		
Qld Fire and Emergency Services (QFES) (No. of employees in QFES: 2200 employees, 2000 Auxiliary, 31,000 rural fire volunteers, and 5,000 State emergency volunteers)	QFES provides for the prevention of, and responses to, fires and other emergency incidents and for rescues services and operations. The key functions of QFES include • to protect persons, property and the environment from fire and hazardous materials emergencies • to protect persons trapped in a vehicle or building or otherwise endangered, to the extent that QFES’s personnel and equipment can reasonably be deployed or used for the purpose	Fire and Emergency Services Act 1990 (current from 3 July 2017 to date)
Western Australia^c^		
Department of Fire and Emergency Services (DFES) (~ 30 employees in the Kimberley and Pilbara regions, and > 1,000 volunteers)	DFES responsibilities include • to carry out functions relating to the provision and management of emergency services • the prevention, control of emergencies and fires; • the protection and saving, and promotion of safety, of life and property endangered by incidents; • the carrying out of rescue operations, search and rescue operations, assistance operations and monitoring activities	Fire and Emergency Services Amendment Act 2012 based on Fire and Emergency Services Act 1998 (and the related Acts—Bush Fires Act 1954 and Fire Brigades Act 1942)
Commonwealth of Australia: Emergency Management Australia (EMA; the Director General of EMA is the Chairperson of the National Emergency Management Committee (NEMC) and its coordinating group, the National Emergency Management Executive Group (NEMEG), and the EMA provides the secretariat for both. The NEMC and NEMEG are the peak consultative forums for emergency management in Australia	EMA guides Australian governments, non-government organisations, EM organisations, agencies and communities in establishing their EM arrangements. It articulates the principles, structures and procedures that support coordination of EM across the nation	Each State and Territory has their own legislation, but key Federal level Acts include i. National Emergency Declaration Act (2020) that recognises and enhances the role of the Commonwealth in preparing for, responding to and recovering from emergencies that cause, or are likely to cause, nationally significant harm; ii. Emergency Response Fund Act (2019) to make arrangements and grants in relation to natural disasters Two main frameworks that guide EM in Australia are i. the National Strategy for Disaster Resilience (the Strategy), adopted by the Council of Australian Governments (COAG) in February 2011; ii. the National Disaster Risk Reduction Framework, 2019 (AIDR 2019). Through the Disaster Recovery Funding Arrangement mechanism, the Australia Government offers financial support to States and Territories in case of emergencies

^a^NTPFES (2019–2020). Northern Territory Police, Fire & Emergency Services 2019–2020 Annual Report. Darwin. NT Government

^b^QFES (2019–2020). Annual report 2019–2020. Queensland Government

^c^DFES (2019–2020). Annual report 2019–2020. Western Australian Government

In remote communities, under existing EM arrangements (Table 1), police officers typically are responsible for managing natural hazards in the first instance, often with limited resources. Typically, community EM plans are kept at the police stations in remote communities, despite the evident reluctance of local community members to frequent those offices given the perception of equivocal experience with police authority (Sithole et al. 2017; Sangha et al. 2019b). Lack of consultation with local community members in the development of EM plans, including planning for prevention, preparedness, or response and recovery from an emergency, is widespread (Sithole et al. 2017; Sangha et al. 2019b). Lack of local community and institutional engagement in the development of detailed response and coordination planning is also lacking at agency level, creating significant frustration for Indigenous residents in remote communities (Sithole et al. 2017; James et al. 2019).

As such, under current arrangements for managing emergencies, it is often the case that EM personnel will travel from a regional centre to remote communities for managing emergencies, applying top-down approaches with little appreciation of local perspectives (Sangha et al. 2019b,c). Existing national, State and Territory EM policy arrangements ignore significant long-term benefits (including financial) which can accrue through supporting local emergency and environmental management capacity, self-reliance and empowerment of local communities, especially given the high frequency of cyclonic, flood and wildfire events across remote northern Australia (NAILSMA 2014; Sangha et al. 2017a, b, 2019c).

### Policy-level implications

Relationships between the State and Indigenous society in northern Australia have historically and generally been problematic and frequently deleterious to Indigenous rights, customs and aspirations (Roberts 2005; Reynolds 2006; Pedersen and Philpott 2019; Scrimgeour 2020). Howitt et al. (2012) argue for a need to address not only the failure to decolonise the lives and territories of Indigenous peoples and the legacies of the colonial past, but also more contemporary failures in intercultural relations and the lack of capacity in State agencies to meet the challenges of cultural diversity. Irrespective of the control by the State, in many communities in northern Australia, Indigenous people continue to exercise some level of traditional cultural self-governance and autonomy. Like Indigenous minorities elsewhere, they often experience threats, risks and hazards that are different to other parts of society (Berkes 1999; Stoffle and Arnold 2003; Whyte 2017; Stacey et al. 2019; Yumagolova et al. 2021). Responses and efforts to adapt to changing circumstances are often hampered by State-constructed hurdles (Howitt 2010). Indeed, Indigenous groups’ social and cultural resilience is often directly undermined by historical and contemporary practices, attitudes and policies of State agencies (e.g. refer Box 1). Consequently, State policies often define Indigenous groups as needy victims or problems, requiring ‘emergency’ or top-down interventions (Lawurrpa et al. 2017). Poor policies and practices, however, extend cycles of colonisation, disempowerment and disengagement, compounding social, cultural and human costs on both indigenous peoples and the wider national society.

In Australia, significant efforts have been underway for over a decade to better match emergency service responses to the needs and capacities of indigenous communities (e.g. CoA 2007a), but the capacity of Australian EM agencies to work with Indigenous communities remains limited. Lessons have sometimes been drawn from past problems (e.g. Brinkley 2009), but implementation of those lessons continues to be difficult to secure in practice (Howitt et al. 2012). Extant Indigenous governance structures stem from and aspire to local cultural connections and knowledge. Genuine engagement of contemporary Indigenous governances by formal government process is rare and typically prescriptive rather than equitable (Howitt 2006; Cross 2008). For emerging significant issues such as the role of climate change in exacerbating natural hazards, this prescriptive approach creates administrative silos under the guise of providing targeted governance for Indigenous Australians (Howitt et al. 2012). Many issues are too complex, however, to be governed by a single agency and require collaborative action by multiple partners—especially given that collaborative action is not easy to implement in situations where there is a mix of resource users, different knowledge systems and power structures, and where the appropriateness of remedial mechanisms are perceived differently by mainstream and Indigenous cultures (Ross and Innes 2005; Berkes 2007; Petheram et al. 2010; Zurba et al. 2012; Whyte 2017; Nursey-Bray et al. 2019; Yumagolova et al. 2021; see Boxes 1, 2). Existing research finds limited opportunity for north Australian remote communities to be actively engaged under current EM frameworks (Morley et al. 2016, Sithole et al. 2017), and the real value of cultural approaches to EM and recovery is rarely acknowledged (Kenney and Phibbs 2014; Sithole et al. 2019a).

## Role of governance and recognition of the expectations and aspirations of remote communities towards developing effective EM partnerships

There is increasing acknowledgement among policy makers and researchers that local knowledge and wide participation from communities are fundamental ingredients of effective EM response (UNISDR 2005; CoA 2007a; Paton and Johnston 2017). Despite this little progress has been made to define what that means in relation to north Australian remote communities and to develop an effective approach (Sithole et al. 2019a, 2021). Personal and institutional understanding of community characteristics, risks, vulnerabilities and capacity help ensure effective response when natural hazards strike and, just as importantly, enhance recovery. In the context of remote Australian Indigenous communities, in the absence of important knowledge about cultural institutions and laws, an emergency situation can become a confused space where time-constrained agencies take expedient options rather than seeking knowledge and advice from the local culturally recognised ‘right people’ (Sithole et al. 2021).

Indigenous communities share strong similarities but are also unique in their composition and situation, allowing for both the design of better approaches to engagement at scale and the need for individual treatment. Research in Galiwin’ku, central Arnhem Land, highlights the confused space where multiple agencies operate and sideline the traditional authority structures (Box 1). Similarly, in nearby Ramingining, research found there was little appreciation by EM agencies of the capability of local community structures, and disconcerting lack of knowledge of their existence (Box 2). When agencies tap into these pre-existing cultural structures, they tap into a network of actors, interests and detailed and nuanced local knowledge systems. They also get access to pathways of decision-making that improve participation, communication and on-ground support. Agencies that ask the right questions and follow-up on the answers will find the challenges of EM in remote communities considerably lighter.

Conversation about partnerships centres around getting the ‘right people’ involved. EM agencies engage haphazardly with communities on the ground and there is a consequent general dissatisfaction with the level and quality of that engagement: who is engaged and what is their local/cultural responsibility, status and capability? Issues of legitimacy are important since communities are not homogenous, but are comprised of multiple cultural diasporas, resettled from homelands elsewhere in the region and often with conflicting interests. Agencies rarely ask communities the questions: “how do you want us to work with you?; how do we connect with your structures?” (Sithole et al. 2019b). In the absence of knowledge and awareness of existing structures and protocols, EM agencies have relied on created structures (e.g. committees) that lack legitimacy locally. Research shows that agencies tend to work with the ‘the easiest, most accessible person’ who is not always the ‘right’ person who might otherwise be identified with the benefit of local knowledge and authority. Further, presuming the authority of people in positions carrying assumed status, benefits and responsibilities alienates the very people they are meant to be informing.

Development of active community participation in EM requires considerable investment in time by agencies, to assess the needs and preferences for engendering appropriate representation. In practice, agencies tend to undercost the time and resources needed to build effective partnerships, tending to the expedience of ticking boxes and reactive engagement. As outlined in the section following, in spite of these big challenges, communities are pressing government agencies for more involvement, more recognition, and greater application of Indigenous knowledges. Although this demand for involvement is sometimes seen as a desire to assume control of the process, community leaders are realistic about what they want—shared, equitable responsibility and resourcing (Box 2).

Research undertaken in Ramingining and Galiwin’ku addressing community resilience in the aftermath of cyclones Lam and Nathan in 2015 reveals strong local views that emergency management (of major hazards) is done for remote community people, not with them (Lawurrpa et. al. 2017). Indigenous community leaders see State interventions, shifting policy settings and the plethora of ethnocentric, poorly engaged service providers as the main agent of erosion in community capability and confidence and therefore as a significant hazard to self-sufficiency (Lawurrpa et. al. 2017; Boxes 1, 2).

Box 1: Galiwin’ku community Participatory Action Research In the months after cyclones Nathan and Lam in early 2015, several senior Yolngu (i.e. local Aboriginal people) were keen to help their community understand and begin to address the ongoing impacts of these two major cyclonic events, with research funding made available through Australia’s Bushfire & Natural Hazards Cooperative Research Centre (BNHCRC). Yolngu leaders ran the project themselves, with logistical and other support provided through NAILSMA. Most Yolngu interviewed in the ensuing participatory action research (PAR) at Galiwin’ku (population ~ 1 100) commented that government emergency teams sent in from the regional capital, Darwin, did a good job restoring services and rendering the community safe and habitable in the aftermath, and that the response was fairly swift and efficient. The natural hazard and corresponding response, however, revealed underlying issues effecting Yolngu authority in their own community, including poor consideration of community members as core players and assets, to engage for assistance with preparation, response and reconstruction.

Community leaders have for many years been concerned at the erosion of their cultural authority and collective agency in the culturally composite settlement of Galiwin’ku. The ‘*Burrumalala—Strong winds*’ research project, commencing in 2015, highlighted this (Lawurrpa et. al. 2017) and inspired collective energy and purpose to resist further fragmentation of leadership and to reassert a structure for clan leader authority at community level. The local PAR enabled broad public recognition of events and processes denuding cultural authority and effective management of their community. Whilst the influences on Yolngu management of their community are highly complex (cultural, economic, historical), the research confirmed a core of issues around colonial agency virtually unanimously expressed by community respondents, including: the paternalism of the mission era; external interventions by the State (e.g. the Northern Territory Emergency Response Intervention in 2007 [see CoA 2007b; Yu et al. 2008]); the imposition of NT local government (Shire) administrations in 2008; unpredictability of financial and service provision over the long term; and an increasing trend by governments to deliver services through uncoordinated, poorly communicated and disengaged approaches.

**Fig. 2:**
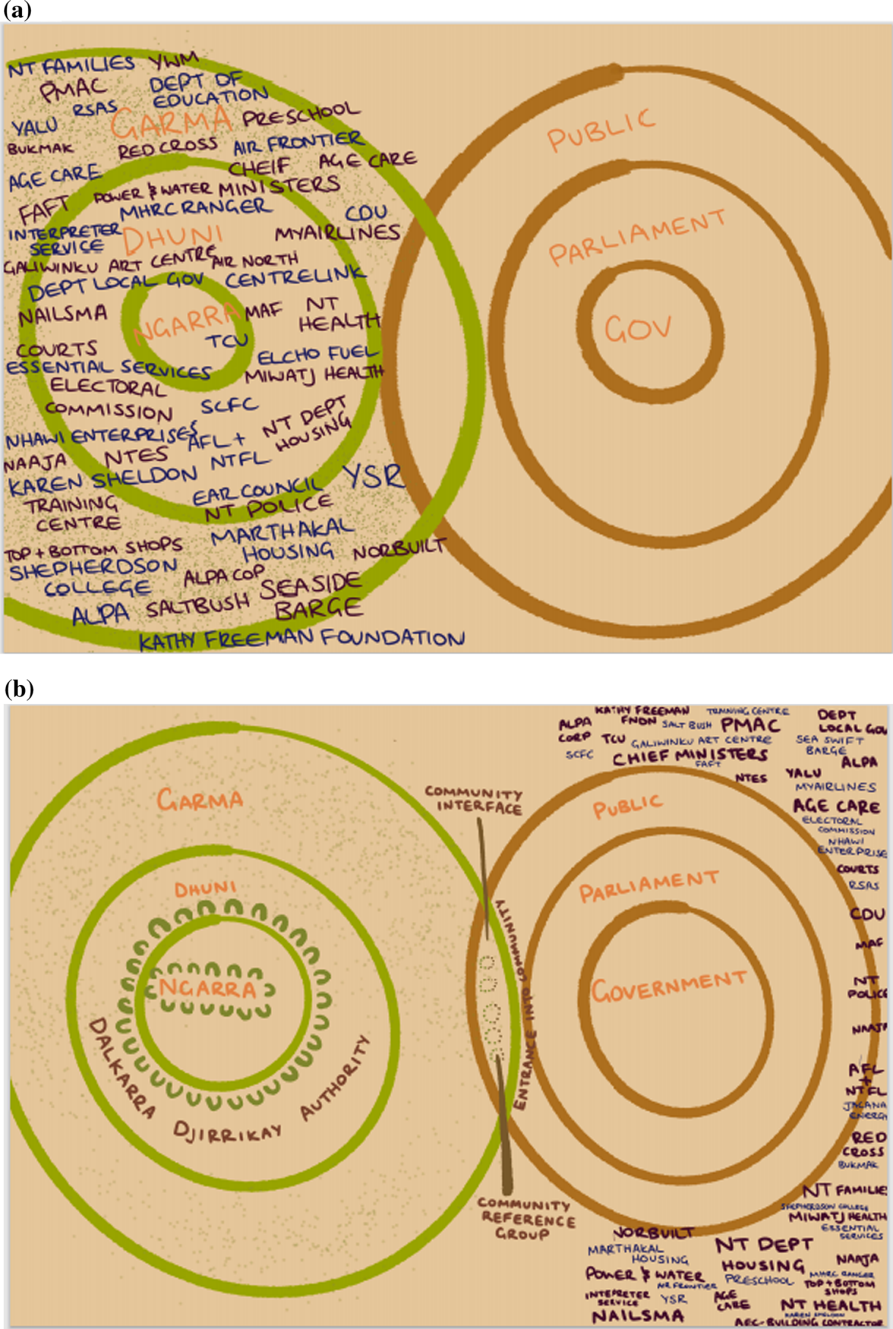

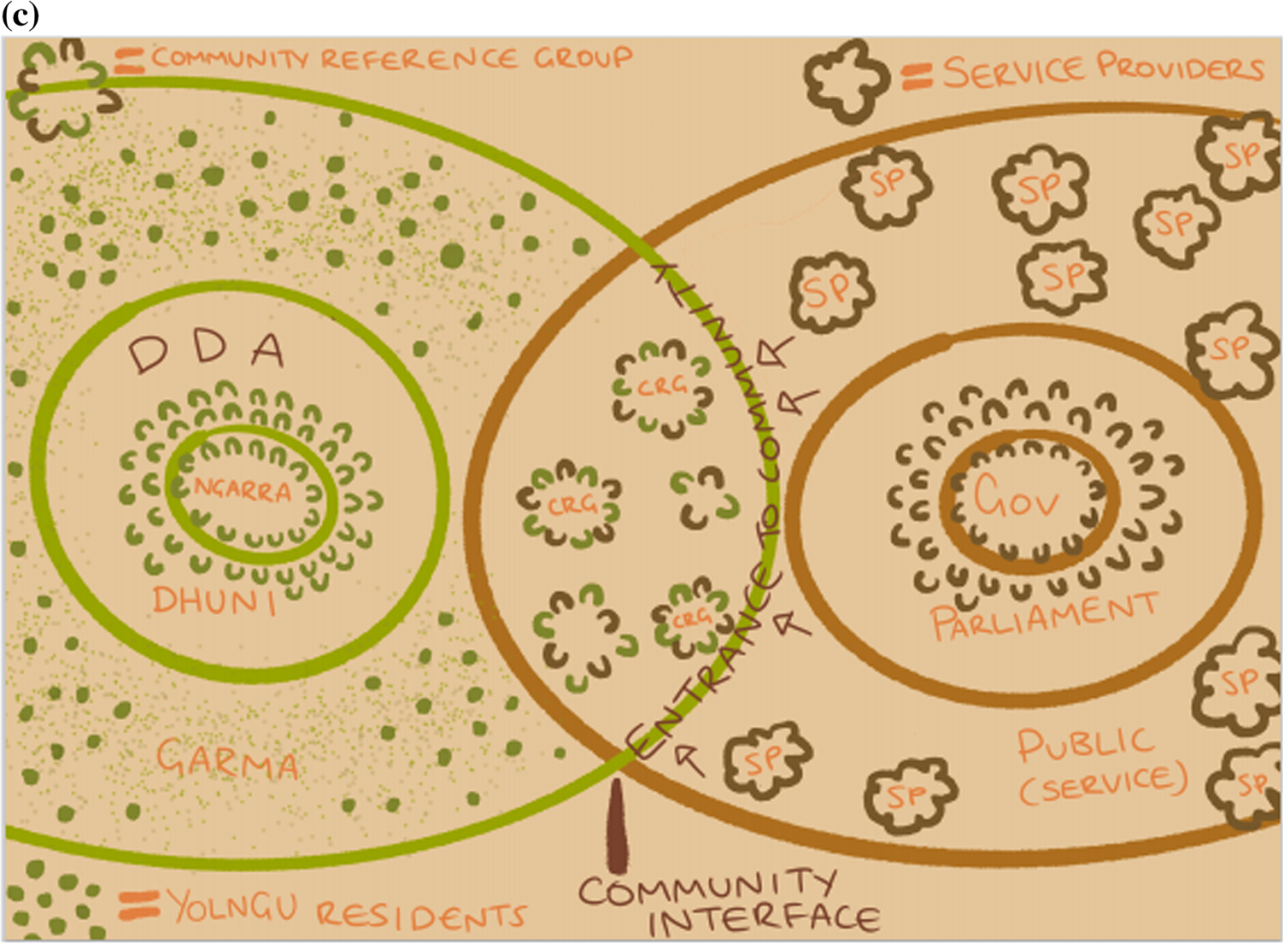
**a** Diagram illustrating the pressure facing Yolngu governance systems from the plethora of services providers wanting or needing to do business at Galiwin’ku. This simplified Yolngu governance sphere is shown to mirror the mainstream governance structure but is virtually unrecognisable under a crowded ‘business as usual’ governance environment. **b** Diagram illustrating the space in which opportunity for better interaction between governance systems exists and where engagement in this cross-cultural space can be more effectively managed and focussed—The Community Interface. The core elements of Yolngu governance are revealed in this picture: Ngarra (the locus of ceremonial leadership and cultural authority); Dhuni (the broader leadership from all clans; Garma (the inclusive Yolngu public organised in lore and culture through the Dhuni). **c** Idealised operation of equitable, two-way, Yolgnu – Balanda (non-Indigenous people), community governance arrangements through mutually appointed Community Reference Groups. Copyright and use of above images (**a**–**c**), with permission by Alan Maratja Dhamarrandji and Ted Gondarra on behalf of the Dalkarra and Djirrikay Authority of Galiwin'ku. Reproduction by Mikaela Earnshaw 2021

The researchers found that EM providers lacked the know-how, the engagement and the intent to ‘build back better’ in support of social and cultural resilience and future capability. The collective realisation of this scenario, amongst the all too familiar maelstrom of poorly engaged service providers (Fig. 2a), stimulated Yolngu researchers to look closely at their own governance capability and develop a strategic plan to engage with EM and other providers in future partnerships.

Community leaders concluded that they needed to reinstate Yolngu authority based in Yolngu law to provide a forum through which EM and other agencies can offer and deliver services more effectively. The structure they set out to recreate (named the Dalkarra and Djirrikay Authority—DDA) is developed independently from non-Yolngu organisations (Fig. 2b). The DDA was a reinvestment into customary authority for Traditional Owners and clan leaders to reclaim credible representation of their community from what they saw as disenfranchising non-Indigenous agency.

The DDA developed to include a secure mandate from the broader community to take responsibility for agreements and partnerships with service providers and agencies such as those in EM. Without such a mandate, credibility and governability of partnerships are seen to be at risk.

DDA members are hopeful that the significant local emotional and other investment in creating the foundation for Yolngu decision-making will, in time, be mirrored by Government agencies and others who, on all accounts, also have significant issues to address in governance, service provision and engagement. The DDA initiative establishes the possibility for more effective engagement with service providers, including EM agencies. The strategy for effective engagement at the interface between community and service providers relies upon the development and management of Community Reference Groups (CRGs) by the DDA for nuanced engagement with service providers relating to their specific agency policies, agendas and grouped activities (such as school education, public housing and community policing). Community residents are represented by identified leaders in these CRGs, having authority and credibility vested by the DDA, and so being responsive to guidance and review by clan leaders (Fig. 2c).

Box 2: Participatory action research in Ramininging Following a BNHCRC-sponsored EM research workshop held at the remote Indigenous community of Ngukurr, population ~ 1100, in 2015 (Morley et al. 2016; Sangha et al. 2017b), participating elders from Ramingining, population 870, felt inspired to initiate discussions concerning EM arrangements with their own community members especially given the significant impacts of Cyclones Lam and Nathan in 2015. Community-based Indigenous researchers used PAR tools (Sithole 2012) to engage with the wider community.

Early consultations with community elders and clan groups described two ways of knowing and doing, the government way and the Yolngu and Bininj (i.e. local Aboriginal people—referred to as Bininj following) way, and that the two were seen to operate independently. EM agencies were observed to need to change from delivering safety, to making it possible for people to be part of securing their own safety (Sithole et al. 2017). Some talked about how agencies pretend they want to work together and want to share responsibility, yet they do not provide spaces for Bininj to be involved.

Bininj concerns go beyond just safety from hazards, it is about resilience of a people, a way of life, a society and their survival into the future. Hazards are seen as evidence of an upset balance between people and country and they are strongly vested in achieving balance. The pillars of Bininj resilience are living on country, engaging with ceremony, language and *Rom* (Bininj Law). People’s presence on country is seen as critical to managing disasters. Bininj maintain that ‘our ways’ are the key to managing disasters and to assure safety especially in remote communities. ‘Our ways’ include the undertaking of specific ceremonies for managing disasters that are held by, and the responsibility of, certain clan groups and need to be performed as a way of managing hazards. ‘Our institutions’ refer to the right institutions, the right people, right holders of knowledge, and the right hierarchy for action before, during and after a hazard. ‘Our practices’ refer to a suite of activities which form obligations an Aboriginal person has for managing country and maintaining balance. These responsibilities don’t disappear because one lives away from *country*. When *country* is neglected, people suffer too, and disasters are an indication to some of an imbalance that must be corrected. ‘Our knowledge’ refers to a huge body of Indigenous experiential knowledge, and *Rom* (Sithole et al. 2017, 2019a).

**Fig. 3:**
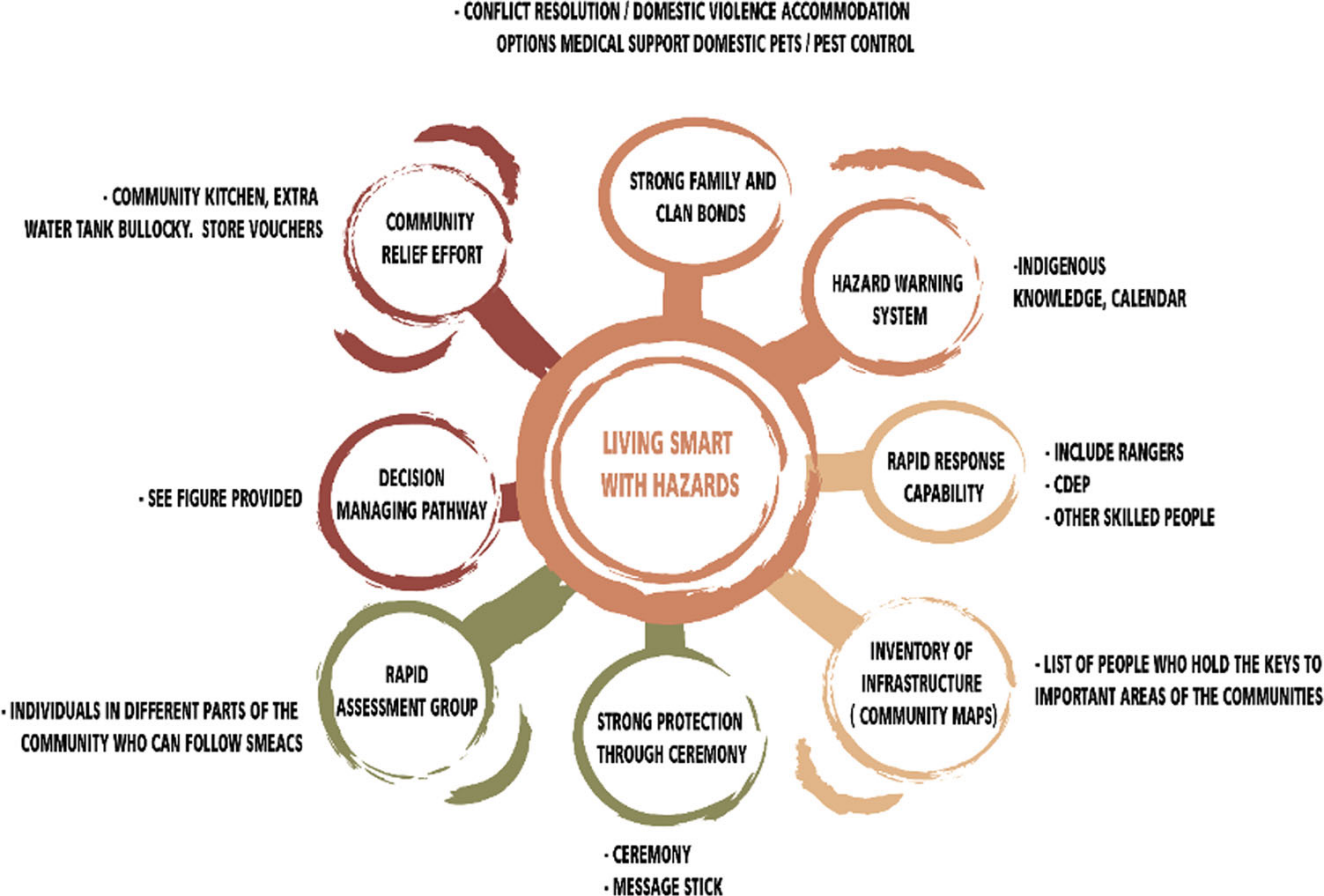
Emergency planning framework as developed by the Ramingining community (after Sithole et al. 2019a, b)

The core issue confronting Ramingining community researchers has been to address “how do we get remote Indigenous communities more effectively involved?” A series of actions were identified by project participants to improve partnerships with, and responsiveness of, EM agencies as follows:

(i) *Develop effective protocols for engaging the community in EM*
  One of the early responses in the project was when the elders and clans sat down through several group discussions and came up with a list of protocols for effective engagement for EM agencies. Although externally developed protocols already exist, these are seen by communities as inadequate. Outside agencies have ignored for too long the lack of alignment between their agency business and Bininj business on country. We need to move the two towards each other. The project produced a detailed list of protocols to guide future engagement with outside agencies for enhancing EM (Sithole et al. 2017, b, 2021).
(ii) *Restore Bininj institutions*
  In the absence of knowledge and awareness of existing structures and protocol, EM agencies have relied on created structures (e.g. committees) that lack legitimacy locally and are there for the convenience of Balanda. These structures have created a negative dynamic in the community that has seen the following:
  Where EM Agencies are engaging with only one person in a community with 14 constituent clan groups, unfortunate dynamics are created. An inclusive structure defined by the community does not need to include all clan groups but does need agreed representation.
  The privileging of individuals by placing them in decision-making and leadership roles that they have no cultural right to be in or desire to play. As well as burdening these individuals, it alienates them from the people they are meant to be informing.
  Ignorance of community, interclan/family dynamics meaning that outside agencies persist with a model where they think an individual can represent all.
  Agencies are unaware of the burden of meetings and the resulting burnout, especially for individuals who sit on multiple committees.
(iii) *Invest in community-driven EM planning*
  Although formal EM community planning documents developed by external agencies exist, the Raminging community had little awareness of plan contents and, in any case, access was restricted since the plan was held in the local police station—typically a location that community members wouldn’t voluntarily visit. Discussions around the plan and what was in it revolved around definitions of community, shared responsibility, working together and walking side-by-side. These crucial issues were important for the community to recast what they wanted to see on the ground. The project developed a new planning framework (Fig. 3). This plan is hosted online and selected members or the community and agencies can add to and edit the plan as required. The plan is open to anyone who has a link and access to an electronic gadget.
(iv) *Building EM capacity on both sides*
  There was recognition that Bininj needed to understand and learn about leadership in emergency situations and to appreciate ‘government ways’ of doing things. As part of allied BNHCRC-funded leadership training research (Sutton et al. 2018), the programme demonstrated a new model for training delivery which underlines the importance of bringing Bininj and Balanda knowledge systems together, focussing on clan-wide training rather than targeted groups, and co-development of training materials. Although the materials were developed with Bininj, they are appropriate also for inclusion of EM agency staff with interests in developing more informed and effective partnerships.

## Exploring solutions for developing effective EM arrangements in remote communities

On the basis of preceding discussion, and in support of the national *Keeping our mob safe strategy* (CoA 2007a), the development of effective EM arrangements serving remote north Australian Indigenous communities needs to address two substantial complementary challenges: (1) provision of cost-effective operational EM services for large numbers of vulnerable remote communities scattered across a vast natural hazards-prone region; and (2) developing supportive EM agency-community partnerships which are respectful and inclusive of local Indigenous knowledges and governance arrangements. We explore solutions to both challenges mindful of their interdependence.

### Volunteerism vs. the contracting of Indigenous Ranger Groups

EM agencies are heavily reliant on delivering services primarily through an unpaid volunteerism model (Table 1). In more densely populated, urbanised regions of Australia, in recognition of significant dwindling volunteer recruitment and retention rates in recent decades, EM volunteer models are undergoing a process of rethinking and transformation with community expectations demanding greater decentralised authority and shared community responsibility, and EM agencies requiring greater accountability and professionalism (McLennan et al. 2016, 2021; Brueckner et al. 2017).

To date, attendant issues confronting volunteerism in remote community settings have received scant attention, despite their evident vulnerability. Traditional volunteerism in remote communities faces significant challenges: consistent commitment to volunteer work is questionable for already relatively impoverished individuals; highly transitory and/or seasonal residency in smaller homeland communities; many community members are unskilled and unemployed; current over-commitment of key individuals; failure to meet police check requirements; lack of drivers licences. In the absence of dedicated EM agency servicing of remote community needs, including long-term funding commitment to training, capacity building and infrastructural resources for developing volunteer services, the great majority of remote communities have no day-to-day capacity for delivering core disaster risk reduction and associated PPRR (Prevention, Preparedness, Response, Recovery) capability (Russell-Smith et al. 2020a).

One of the approaches increasingly emerging throughout Australia, especially in urbanised centres, is the retention of trained, part-time (‘on-call’), remunerated auxiliaries (or ‘retained volunteers’) to augment professional fire and emergency management personnel in times of need (e.g. NTFRS 2018; QFES 2021). The engagement of paid auxiliaries affords an example which, by extension as a fee-for-service model, could usefully serve in remote locales—especially, as considered here, when extending the capacity and remit of the large network of Indigenous Ranger Groups (IRGs) already operative across remote northern and regional Australia (Russell-Smith et al. 2020a).

As at March 2021, there were 129 IRGs nationwide, employing 900 full-time rangers funded by the Australian Government’s Indigenous Ranger Programme (NIAA 2021). In addition, the Queensland Government supports a further 24 IRGs, employing over 100 full-time positions through their Indigenous Land and Sea Ranger Programme (QDES 2021). The great majority of these IRGs and available positions are located in remote northern regions, as well as servicing vast tracts of the remote desert rangelands in central Australia. Although the contracted focus of IRGs is to meet a variety of biodiversity and cultural resource management targets set by Government (Kerins 2012; Hill et al. 2013), such groups could realistically deliver, if roles were formally expanded and appropriate training and resourcing provided, fire prevention and broader EM services to an extended number of remote communities and wider landscape settings (Sangha et al. 2017a, b, 2019b; Russell-Smith et al. 2020a). A salient example is afforded by the actions of a group of north Queensland IRGs (Girringun, Carpentaria Land Council, Ewamian, Yuku Baja Muliku) in the immediate aftermath of Cyclone Yasi in 2011, a major Category 5 event causing, as noted previously, AUD 1.4B in structural damage. These IRGs provided sustained voluntary assistance to the battered north Queensland Cardwell community, winning widespread appreciation, demonstrating the capacity and capability of IRGs to be effective first respondents, and underscoring the need for developing a broader Indigenous Emergency Response framework (Archer 2019).

Significant progress with the development of the IRG programme has been made since inception of Australia’s National Reserve System *Indigenous Protected Area* programme in 1997, and substantial expansion of IRGs under the *Working on Country* programme from 2007. These programmes are publicly funded and, despite strong community support, policy commitments have proven to be haphazard (e.g. Altman and Kerins 2012; Morrison et al. 2019). In response, some IRGs have necessarily sought to diversify their contracted core cultural and natural resource management responsibilities to include a range of complementary fee-for-service activities: cultural heritage survey and management, feral animal and weed management, fire management, biosecurity assessments, coastal surveillance and quarantine, infrastructure site maintenance, fencing and track maintenance, minesite rehabilitation and monitoring, research support services (CoA 2018).

One particularly prospective opportunity for IRGs operating in fire-prone north Australian savannas has been the undertaking, since the mid-2000s, of landscape-scale, market-based savanna burning greenhouse gas emissions reduction projects (Russell-Smith et al. 2013; Edwards et al. 2021). As at 2020, savanna burning projects on Indigenous lands collectively covered ~ 180,000 km^2^ abating ~ 2.8 MtCO_2_-e annually, earning AUD 40 m per yr (Sangha et al. 2020). The emergence of savanna burning as an industry sector illustrates the potential for IRGs developing contracted linkages with EM institutions to undertake management services around remote communities, and townships and infrastructure more broadly (Russell-Smith et al. 2020a). Additionally, the undertaking of prescribed early dry season fire management provides other ecosystem services opportunities, including development of incentivized schemes for delivering land condition, water quality and biodiversity conservation outcomes both at landscape scales (e.g. reducing contemporary extensive wildfire impacts), and for targeted applications (e.g. management of appropriate habitat conditions for fire-vulnerable flora and fauna). While development of such schemes is still in its infancy, Russell-Smith and Sangha (2018) outline some of the considerable advances already being undertaken towards the development of ecosystem services opportunities in north Australian savanna environments.

For the most part, however, fee-for-service opportunities continue to be restricted, with little recognition of the potential for, or dedicated investment provided by, responsible government authorities to build the business and associated governance capacity of community-based IRG institutions (Russell-Smith et al. 2020a; Sangha et al. 2020). Such capacity is critical if remote communities are to emerge from current levels of welfare dependency, and associated social and economic deficit and disadvantage (COAG 2011; CoA 2020). Indeed, Indigenous-owned local enterprise is widely seen as the vehicle for redressing post-mission era dependencies (Morrison 2012; James et al. 2021). As illustration, based on 2016 census data (ABS 2016), Sangha et al. (2020) describe employment characteristics in two typical Northern Territory remote communities, Borroloola (population 871, 77% Indigenous) and Maningrida (population 2612, 91% Indigenous), where 60% and 70%, respectively, of the eligible Indigenous population workforce was unemployed. Such employment opportunities as exist occur predominantly in the government service sector (e.g. teaching, health, administration).

Importantly, as illustrated through formal scenario planning activities undertaken as part of the broader BNHCRC research programme reported here, IRG members have demonstrated considerable appetite for engaging with and delivering EM services under a contracted fee-for-service model (Box 3). Recognising that implementation of enhanced partnership engagement as identified under Scenario 2 (Box 3) will incur significant recurrent resourcing issues for Government, in Box 4, we present an illustrative financial model inclusive of costs for part-time employment of two Indigenous Rangers plus realistic operational requirements, and associated cost savings to government (following Sangha et al. 2019a), for effective EM resourcing of all current IRGs in north Australia occurring north of 20° S.

**Fig. 4:**
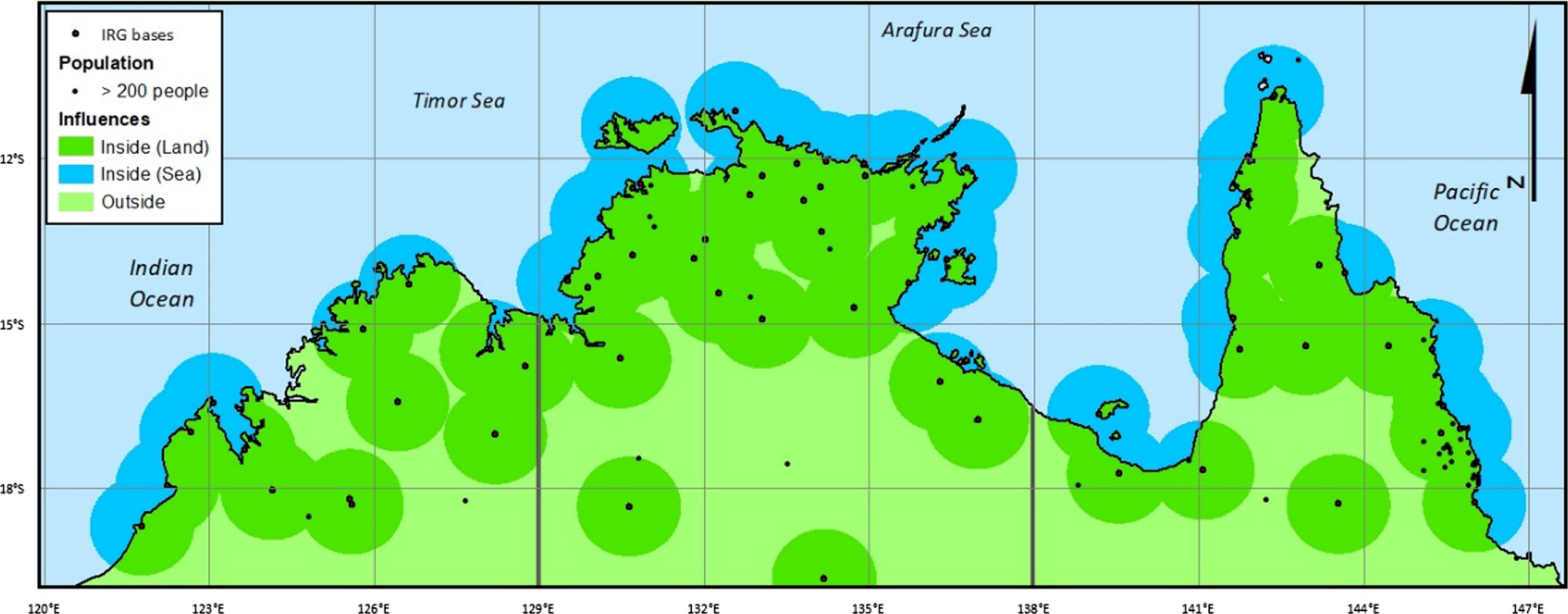
Locations of remote communities with > 200 persons and indicative sphere of influence of Indigenous Ranger Groups defined as a 100 km radius buffer around each ranger base, in north Australia (north of − 20° S). Sources: https://apps.des.qld.gov.au/land-sea-rangers/; https://www.niaa.gov.au/sites/default/files/files/ia/IEB/ipa-national-woc-map-mar-2021.pdf. Note that the map omits island 14 IRGs located in the Torres Strait, between mainland Australia and Papua New Guinea

Application of this financial model example illustrates that building the EM capacity of IRGs can potentially afford substantial net fiscal benefits to government (Box 4), in addition to broader employment, social, community and effective EM delivery benefits. Further, assuming that each IRG can effectively provide EM engagement and services within an operational radius of 100 km of their administrative base, the distribution of current IRGs funded through Australian and Queensland Governments would deliver enhanced EM service capacity for all but three Indigenous communities with > 200 residents (ABS 2016) across our north Australian focal region (Fig. 4). We acknowledge however that, and as recommended by James et al. (2021), this illustrative model requires further detailed financial analysis, including for requisite training and community education activities, to explore the efficacy of implementing cost-effective EM approaches appropriate for diverse remote community settings.

Box 3: Testing the interest of Indigenous Ranger Groups for undertaking contracted EM services Assessment of the prospective interest of IRGs from across the Northern Territory with the undertaking of EM activities was conducted as part of formal scenario planning activities (refer Sangha et al. 2019b for methods) involving three remote communities from central (Boroloola) and southern locations (Hermannsburg, Yuendumu), and participants at two leadership training workshops from coastal and sub-coastal northern regions involving, respectively, 29 Indigenous rangers representing six IRGs (Surjan et al. 2019), and 22 Indigenous rangers representing 10 IRGs (Edwards et al. 2020). All communities involved experience major natural hazards including recurring wildfire, and to some extent major flooding, whereas destructive cyclones typically impact only coastal communities.

**Fig. 5:**
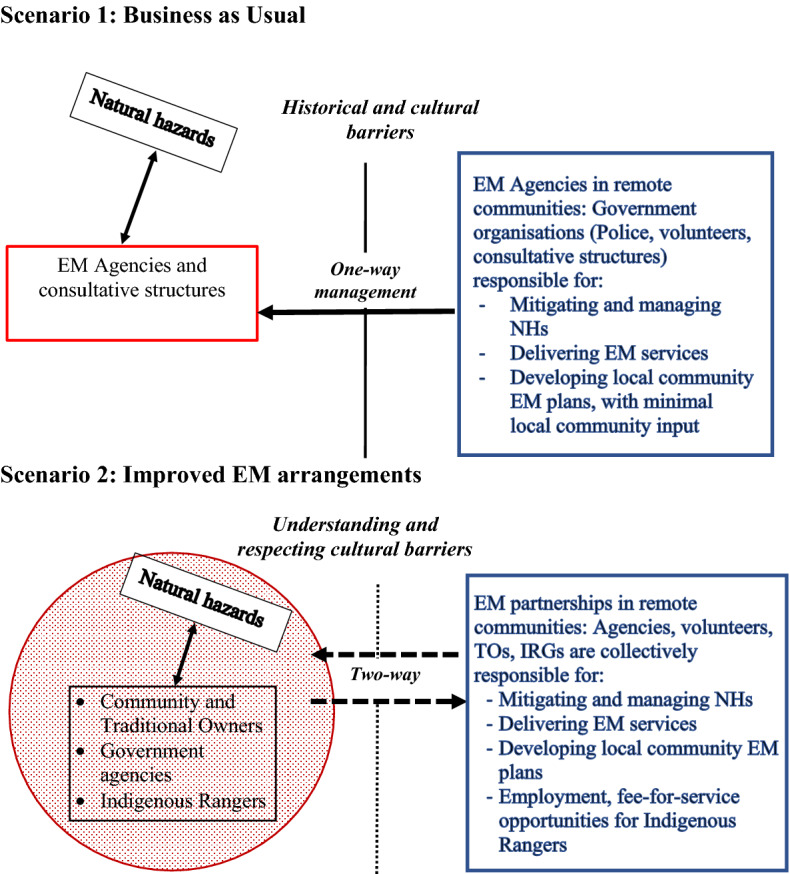
Generalised summary of formal scenario planning exercises with Indigenous Ranger Groups and remote community members in the Northern Territory, addressing two feasible EM scenario outcomes

Two EM engagement scenarios were explored with IRG and remote community members in respective scenario planning exercises, where participants were asked to address feasible EM arrangements in five years time under: Scenario 1—business-as-usual; Scenario 2—improved EM arrangements. The stark engagement contrast between these two scenarios as perceived by participants is summarised in Fig. 5. Notably, the ‘improved EM arrangements scenario’ was envisaged as emphasising both respectful two-way partnerships between EM agencies with remote Indigenous communities (refer Boxes 1, 2), and providing direct employment and contractual opportunities for IRGs.

Box 4: Costs and benefits associated with enhanced engagement arrangements with Indigenous Ranger Groups As an illustration of economic benefits associated with enhancing EM capacity and engagement with remote communities through providing additional support for IRGs, we estimated costs, and associated cost savings to government applying the Avoided Cost method (following Sangha et al. 2019a), for IRGs in north Australia occurring north of 20° S (refer Fig. 4). For calculating costs, we considered employment of two half-time EM engagement positions, comprising one male and female ranger, per IRG. Separate male and female representation is required in line with Indigenous work-cultural protocols where typically women and men, from different cultural moieties, have respective responsibilities (Sithole et al. 2017). Applied standard employment costs were AUD 71 000 per yr for two half-time rangers, and operational costs associated with purchase of a suitable 4WD (AUD 70 000, amortised over 5 years) and fuel and maintenance (AUD 30 000 per yr), to facilitate community engagement and associated EM activities.

To estimate cost savings, we used State/Territory specific welfare expenditure data from the Indigenous Expenditure Report (2015–16) included in SCRGSP (2017) where, following Sangha et al. (2021), conservatively we applied 0.5 of the total expenditure per person that involvement in meaningful work opportunities saves government expenditure on welfare payments through enhancing economic participation, health, reducing domestic violence and incarceration rates, and offering a safe and secure environment in communities. We accounted for associated costed benefits per position including values for pride and self-esteem, reducing domestic violence, and incarceration rates, derived from SVA (2016), SCRGSP (2017), and Sangha et al. (2019a).

Costs for employing two half-time EM rangers from each IRG were estimated at AUD 4.26 M per yr, and annualised operational costs were estimated as AUD 2.64 M per yr—with an estimated total cost of AUD 6.9 M per yr (Table 2). Conversely, benefits to government associated with creation of these Indigenous EM positions were calculated conservatively at AUD 9.1 M per yr (Table 2), taking into account expenditure costs associated with social welfare payments, domestic violence, incarceration and loss of self-esteeem (Hamburger et al. 2016; SVA 2016; ALRC 2017; PWC Indigenous Consulting 2017; SCRGSP 2017). In addition, it is well recognised that IRG employment opportunities have significant health benefits (Burgess et al. 2005), uncosted here.

**Table 2:** Estimated costs, and cost savings to government, associated with building the EM service capacity of all north Australian IRGs situated north of 20° S (all $ values in 2020 AUD)

	NT	Qld	WA
(a) Costs			
Number of IRGs	31	17	12
1. Cost of employing two EM personnel (1 male and 1 female) half-time per IRG @ $71,000 per yr	2 201 000	1 207 000	852 000
2. Cost of a vehicle (@$70,000) for each IRG, amortised over 5 years	434 000	238 000	168 000
3. Operational costs (@$30 000 per yr)	930 000	510 000	360 000
Total costs	3 565 000	1 955 000	1 380 000
	6 900 000		
(b) Cost Savings for main benefiting sectors where benefits generated from EM-related employment in remote communities. Note that cost-savings below apply to an ‘average’ person and do not relate to individuals potentially employed in EM programmes			
1. Welfare cost savings (Applying 0.5 average welfare costs for Indigenous people, i.e. in the NT $36 297/person/yr, Qld $21 503/person/yr; and WA $27 730/person/yr; source: Indigenous Expenditure Report 2015–16, in SCRGSP (2017)	2 250 414	731 119	665 520
2. Pride and self-respect ($15 141/person/yr; source: SVA (2016)	938 742	514 794	363 384
3. Domestic violence-related cost savings ($20 950/person/yr; source: SVA (2016)	1 298 900	712 300	502 800
4. Incarceration-related cost savings (average cost of $8978/person/yr; source: Indigenous Expenditure Report 2015–16, in SCRGSP (2017)	556 636	305 252	215 472
Total cost savings for each State/Territory ($ per yr)	5 044 692	2 263 465	1 747 176
Total savings ($ per yr)	9 055 333

### Effective EM agency-community partnerships

For all their potential, the role of IRGs in assisting more effective delivery of EM services needs to be balanced by an understanding of and commitment to supporting appropriate community governance structures and arrangements in respective communities. Whereas IRGs invariably have strong ties to traditional land owners (i.e. ‘Traditional Owners’) and clan leaders and thereby have mandates to act, seek advice, provide direction and relevant decision-making, they are not necessarily the first nor most appropriate point of engagement for EM and other agencies (Boxes 1, 2). As described for the Galiwin’ku community (Box 1), traditional governance authorities such as the Dalkarra and Djirrikay Authority (DDA) can provide an appropriate portal for first transacting arrangements and linkages between government agencies, other service providers, and appropriate community members and institutions, including IRGs. Additionally, many IRGs are hosted and administered by regional Land Councils representing the land (and sea) rights and interests of Indigenous title holders and claimants, but which typically possess limited capacity to support development of autonomous businesses and the local aspirations of community-based IRGs. Such organisations can, however, assist with providing guidance to EM agencies for identifying appropriate Elders and Traditional Owners with senior community leadership roles and customary responsibilities.

Effective engagement with customary Indigenous community governance institutions and individuals is recognised as a core principle of current EM policy and practice internationally (UNISDR 2005) and in Australia (CoA 2007a)—although implementing inclusive intercultural delivery in both settings evidently continues to provide significant challenges (e.g. Howitt et al. 2012; Sangha et al. 2017b, 2019b; Sithole et al. 2019a, 2019b; Stacey et al. 2019; Yumagolova et al. 2021). Australia’s *Keeping our mob safe* remote Indigenous community emergency management strategy states that “the development of effective partnerships between remote Indigenous communities and emergency-related agencies is the key to success of this strategy” (CoA 2007a, p. 7), and goes on to outline key supportive implementation priorities, including (1) recognising Indigenous community decision-making structures; (2) developing effective engagement relationships with community leaders; (3) co-development and -ownership of community EM plans, including recognition of traditional knowledge and experience; (4) identifying and making resources available to meet EM infrastructure, essential services, education, training and ‘possible employment’ needs.

Documented evidence of remote community EM experience presented here (especially Boxes 1–3) indicates the limited extent to which these affirmative national engagement principles have been met to date. We note however that at State and Territory jurisdictional level, some significant progress has been occurring, for example, in the Gulf of Carpentaria region, Queensland, IRGs hosted by the Carpentaria Land Council Aboriginal Corporation (CLCAC) have been providing successful front-line EM services in partnership with Queensland EM agencies for over a decade; in the Northern Territory, from the late 1980s, the rural fire management agency Bushfires NT has provided substantial inclusive practical assistance both to pastoral and Indigenous stakeholders; and in the Kimberley region of northern Western Australia, the Department of Fire & Emergency Services (DFES) has been providing long-term infrastructure and mentoring support towards developing two remote community EM volunteer brigades.

While acknowledging the significant intercultural, logistical and resourcing challenges involved, in order to further support their statutory obligations, we encourage responsible EM regional agencies and national institutions to consider the practical, financial and broader community development benefits (e.g. Box 4) which can accrue from effective partnership relationships with receptive Indigenous communities.

## Conclusion

Remote Indigenous communities across northern Australia face significant natural hazards challenges, particularly the impacts of markedly increasing dry season temperatures and associated wildfire risks, and potentially much more severe cyclonic events. Despite the currency of Australian national policy that espouses development of inclusive EM arrangements in remote Indigenous communities, limited steps have been taken to date to realise effective implementation. Similar intercultural legacy and institutional barriers and challenges face disempowered and marginalised Indigenous and local communities in other international settings.

Building on a seven-year collaborative intercultural socio-environmental and policy research undertaking directly involving remote community members, researchers and agency staff, we have focussed on investigating the EM challenges and aspirations as perceived by community members, and explored opportunities for effecting supportive and sustainable EM agency-community partnerships. Whilst acknowledging the significant logistical and resourcing challenges associated with developing inclusive and foundational governance partnerships given the diversity of remote community contexts, we demonstrate that (a) the potential for cost-effective delivery of contracted EM services to many remote communities is already achievable through the geographically expansive network of existing Indigenous Ranger Groups, and (b) such engagement can also serve as an instructive model for building economic capacity, enterprise and employment opportunity in remote communities where little currently exists. We acknowledge further that such a vision will take time to realise, but recognise that (c) positive regional examples of agency-community collaborations are already in train, (d) as illustrated here many communities and their respective IRGs are keen to participate where opportunity presents, and (e) there is evident commitment and enthusiasm on the part of both engaged senior EM managers and community leaders to further explore collaborative partnership models (James et al. 2021).

Our synthesis aims to serve ongoing empowerment of Indigenous remote communities in, and policy support for, collaborative natural hazards management arrangements across northern Australia. We trust that the experience reported here can also help inform the development of collaborative EM arrangements in other intercultural settings.
